# Exploring the depths of light: a conversation with Professor Chris Xu

**DOI:** 10.1117/1.NPh.12.3.030402

**Published:** 2025-09-25

**Authors:** Tianyu Wang

**Affiliations:** Boston University, College of Engineering, Boston, Massachusetts, United States

## Abstract

Professor Chris Xu reflects on his journey from a curious student in China to a leading researcher at Cornell University, offering insights into the evolution of his work, the mentors who shaped him, and the future of brain imaging.

**Figure:**
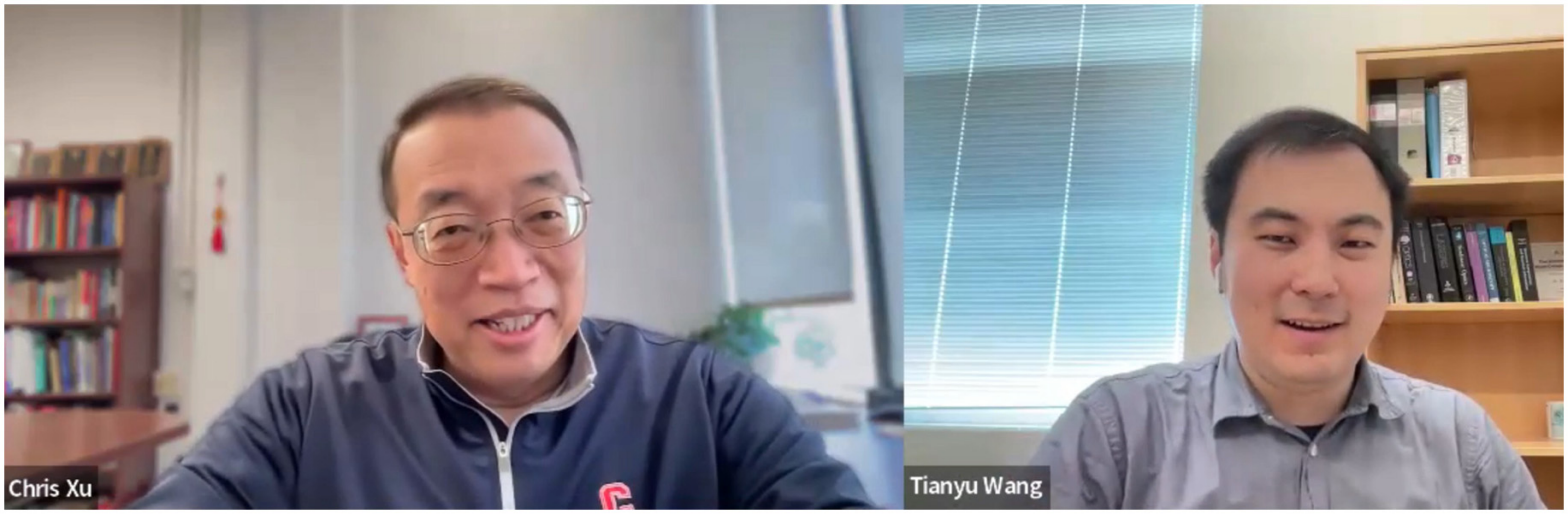
Tianyu Wang (right) interviewed Chris Xu (left), Professor of Engineering at Cornell University, Director of Cornell Neurotech Engineering, and Director of the School of Applied and Engineering Physics. View the interview video at https://doi.org/10.1117/1.NPh.12.3.030402.

In the world of optical microscopy, Professor Chris Xu is widely considered a pioneer in deep tissue imaging. Xu has helped shape the field through innovations like long-wavelength two-photon microscopy, three-photon microscopy, and simultaneous spatial-temporal focusing.

In a recent wide-ranging conversation Xu reflected on his journey from a curious student in China to a leading researcher at Cornell University, offering insights into the evolution of his work, the mentors who shaped him, and the future of brain imaging.

Xu’s fascination with optics began in Professor Watt Webb’s lab at Cornell, where he encountered lasers for the first time. “Optics always gave me the sense that it’s like walking into a toyland,” he recalls. “You can tinker, have ideas, and make them happen.” That spirit of improvisation and creativity became a hallmark of his approach to research.

One formative experience was a two-photon microscopy demo at the Marine Biology Lab in Woods Hole. Despite technical setbacks, Xu and his team improvised using a ZEISS confocal system and ultimately won an award. “That was trial by fire,” he says. “Afterwards, I felt like a senior graduate student.”

## Mentorship and Motivation

Xu credits much of his early development to Webb’s mentorship. “Meeting Professor Webb was the biggest break in my life,” he says. Webb’s ability to communicate complex ideas and energize his students left a lasting impression. “Every time I left his office, I felt more energized than when I went in.”

While Webb’s technical background was in material science, Xu admired his visionary thinking. “He had the ability to identify and focus on what’s really important,” Xu says, a skill Xu continues to emulate.

## Bell Labs: a Crucible of Excellence

After his PhD, Xu joined Bell Labs, where he worked with luminaries like Winfried Denk and David Tank. “It was intimidating at first,” Xu admits. “Everyone was an expert, writing textbooks.” But the environment was rich with opportunity: “You could walk a few flights of stairs and find a world expert in any field.”

Xu describes Bell Labs as a place where researchers were both generals and soldiers—expected to generate ideas and execute them independently. “That dual role taught me a lot,” he says.

## Returning to Academia: Bridging Fields

When Xu returned to Cornell, he initially focused on telecom systems. But he soon realized that applying telecom technologies to biomedical imaging offered a unique advantage. This pivot led to three major projects: spatial-temporal focusing, long-wavelength imaging, and frequency-multiplexed imaging. Of these, long-wavelength imaging proved most effective for deep tissue applications.

## Three-Photon Microscopy: a Potential Game-Changer

Xu’s interest in three-photon microscopy began during his postdoc years but gained momentum around 2009. “It was an obvious path forward,” he says, describing a back-of-the-envelope calculation that revealed its potential for deeper imaging and better signal-to-background ratio. Despite technical hurdles, the approach became a cornerstone of his lab’s work.

## Looking Ahead: the Future of Brain Imaging

Asked to predict the next 20 years in brain imaging, Xu emphasizes continued progress in depth, speed, and resolution. He also sees AI playing a larger role—not just in data analysis but in hardware development. “AI will infuse into all aspects of technology,” he says.

Another key enabler will be probe development. “GCaMP indicators transformed what we could do,” Xu notes. “Imagine what a future GCaMP12 could unlock.” He also dreams of discovering dyes with massive cross-sections, a breakthrough that could revolutionize nonlinear imaging.

Looking 50 years ahead, Xu envisions more interdisciplinary research institutes. “Breakthroughs often happen at the intersection of fields,” he says. He hopes future institutions will blend the bottom-up creativity of universities with the mission-driven focus of industrial labs.

As our conversation wrapped up, Xu reflected on the importance of curiosity, collaboration, and resilience. Whether improvising in a lab or navigating career transitions, his journey underscores the power of combining vision with technical rigor.

“It’s been a happy ride,” he says. And for the field of neurophotonics, it’s clearly a ride that is far from over.

